# MicroRNA-Mediated Gene Silencing in Plant Defense and Viral Counter-Defense

**DOI:** 10.3389/fmicb.2017.01801

**Published:** 2017-09-20

**Authors:** Sheng-Rui Liu, Jing-Jing Zhou, Chun-Gen Hu, Chao-Ling Wei, Jin-Zhi Zhang

**Affiliations:** ^1^State Key Laboratory of Tea Plant Biology and Utilization, Anhui Agricultural University Hefei, China; ^2^College of Horticulture and Forestry Sciences, Huazhong Agricultural University Wuhan, China; ^3^Key Laboratory of Horticultural Plant Biology (Ministry of Education), College of Horticulture and Forestry Sciences, Huazhong Agricultural University Wuhan, China

**Keywords:** defense, counter-defense, gene silencing, miRNA, virus

## Abstract

MicroRNAs (miRNAs) are non-coding RNAs of approximately 20–24 nucleotides in length that serve as central regulators of eukaryotic gene expression by targeting mRNAs for cleavage or translational repression. In plants, miRNAs are associated with numerous regulatory pathways in growth and development processes, and defensive responses in plant–pathogen interactions. Recently, significant progress has been made in understanding miRNA-mediated gene silencing and how viruses counter this defense mechanism. Here, we summarize the current knowledge and recent advances in understanding the roles of miRNAs involved in the plant defense against viruses and viral counter-defense. We also document the application of miRNAs in plant antiviral defense. This review discusses the current understanding of the mechanisms of miRNA-mediated gene silencing and provides insights on the never-ending arms race between plants and viruses.

## Introduction

Viruses are among the most important causal agents of infectious diseases in both animals and plants. Disease symptoms associated with viral infection in plants include stunting, yellowing, mosaic patterns, ringspot, leaf rolling, wilting, necrosis, and other developmental abnormalities (Wang et al., 2012). During the course of evolution, plants have employed versatile mechanisms against invading viruses, such as RNA silencing, hormone-mediated defense, immune receptor signaling, protein degradation and regulation of metabolism (Calil and Fontes, 2016). Evidence is accumulating that RNA silencing plays critical roles in plant immunity against viruses. RNA silencing, which is induced by small RNAs (sRNAs), is a central regulator of gene expression and an evolutionarily conserved mechanism in eukaryotic organisms (Eamens et al., 2008; Pumplin and Voinnet, 2013). Plant sRNAs are grouped into two major classes: microRNAs (miRNAs) and small interfering RNAs (siRNAs). Plants have evolved three basic RNA silencing pathways, which are represented by the miRNA pathway, the siRNA-directed RNA degradation pathway, and the siRNA-directed DNA methylation (RdDM) pathway (Baulcombe, 2004; Eamens et al., 2008; Wang and Smith, 2016).

MicroRNAs are endogenous RNAs of 20–24 nucleotides that are processed by Dicer-like (DCL) proteins from imperfectly paired hairpin precursor RNAs, and typically targeting a single site in their target mRNA (Voinnet, 2009; Axtell, 2013). siRNAs are similar sized and also require DCL proteins for biogenesis, but they are derived from perfectly paired double-stranded trigger RNA molecules that can be endogenous or derived from introduced RNAs, transgenes, or viruses, affecting multiple sites on the target RNA (Bartel, 2004, 2005). The siRNA-mediated gene silencing serves as a general defense mechanism against plant viruses (Wang et al., 2012; Pumplin and Voinnet, 2013; Revers and Nicaise, 2014; Ghoshal and Sanfacon, 2015; Khalid et al., 2017), while miRNAs are involved in plant growth and development, signal transduction, protein degradation, and response to biotic and abiotic stresses (Voinnet, 2009; Zhang et al., 2012; Bologna and Voinnet, 2014). However, miRNAs also play critical roles in plant–virus interactions (Li et al., 2012; Ramesh et al., 2014; Tiwari et al., 2014; Ghoshal and Sanfacon, 2015; Huang et al., 2016). Nowadays, miRNA-mediated gene silencing has been applied to protect several agricultural crop species against infection by diverse viruses (Tiwari et al., 2014; Khalid et al., 2017). In this review, we (1) document the biogenesis and origin of miRNAs and the current understanding of miRNA-mediated gene silencing mechanism in plants; (2) describe the roles of miRNAs in plant–virus interactions; and (3) discuss the current applications of miRNA-mediated gene silencing and advances in the technique in plant science.

## Origins, Biogenesis and Modes of Action of Plant miRNAs

**FIGURE 1:**
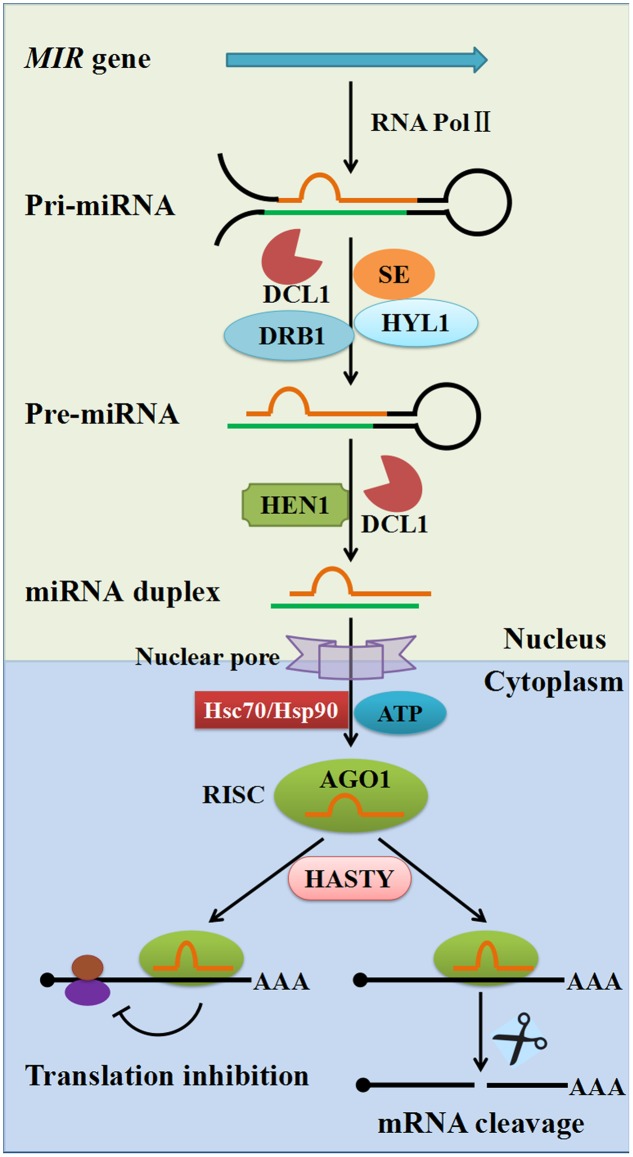
The biogenesis and regulation mechanisms of plant miRNAs. Plant pri-miRNAs are mostly produced from *MIR* genes by RNA polymerase II (Pol II). Pri-miRNAs are cleaved into pre-miRNAs by DCL1 with the assistance of SE, dsRBP and HYL1. Pre-miRNAs are further processed into 21–24 nucleotide duplex miRNAs by the combined action of DCL1 and HEN1. Duplex miRNAs are methylated by HEN1 into mature miRNA duplexes and are exported to the cytoplasm through the action of plant exportin 5 ortholog HASTY. The guide-strand (red) is then loaded onto an AGO protein with the help of Hsc70/Hsp90 chaperone and ATP, followed by passenger strand (green) ejection, to form a RISC. There are two modes of plant miRNA action in cytoplasm: in one, the miRNA regulates its target at the protein level through translational inhibition (left); in the other, the miRNA regulates its target at the mRNA level through mRNA cleavage (right). AGO1, Argonaute 1; DCL1, Dicer-like1; SE, Serrate; HEN1, Hua enhancer1; DRB1, Double-strand RNA binding protein1; HYL1, Hyponastic leaves1; RISC, RNA-induced silencing complex.

miRNAs are derived from single-stranded RNA transcripts (*MIR* genes) that can fold back onto themselves to produce imperfectly double-stranded stem–loop precursor structures. The mechanisms of miRNA biogenesis and modes of action are well-established in plants (**Figure 1**). The *MIR* genes are RNA polymerase II (Pol II) transcription units that produce the primary miRNA transcript (pri-miRNA), which is then cleaved by DCL1 in the nucleus, leading to production of the shorter precursor-miRNA (pre-miRNA, partially duplex molecule with a single-stranded loop, mismatches, and a single-stranded extension) with the assistance of the dsRNA-binding protein 1 (DRB1) and HYPONASTIC LEAVES1 (HYL1). Subsequently, the miRNA duplex (miRNA/miRNA^∗^ where miRNA^∗^ stands for the passenger strand) is released from the pre-miRNA stem–loop structure by the second cleavage step with the help of the combined action of DCL1 and HYL1. The mature miRNA duplex is methylated by the sRNA-specific methyltransferase HUA ENHANCER1 (HEN1) and then exported to the cytoplasm through the action of the plant Exportin-5 ortholog HASTY and other unknown factors. In the cytoplasm, the mature miRNA strand is loaded onto Argonaute 1 (AGO1) to form an RNA-induced silencing complex (RISC) with the help of Hsc70/Hsp90 chaperone and ATP, followed by the passenger strand ejection (Iwasaki et al., 2010; Nakanishi, 2016). The RISC then uses the miRNA to guide the slicer activity of AGO1 to repress the expression of complementary target mRNAs (Llave et al., 2002). Two main modes of action have been described for target repression caused by miRNAs: translational repression and cleavage of target mRNA. It is worth noting that animal miRNAs bind 3′ untranslated regions (UTRs) and function predominantly through translational repression; whereas plant miRNAs primarily target the coding regions of mRNA, and repression of gene expression is mostly by transcript cleavage. Nevertheless, recent studies have indicated that miRNA-mediated translational repression is also commonly found in plants (Brodersen et al., 2008; Djuranovic et al., 2012; Iwakawa and Tomari, 2013; Li et al., 2013).

The first miRNA (lin-4) was discovered in *Caenorhabditis elegans* (Lee et al., 1993), and a large number of miRNAs have since been identified in animals and plants. Initially, miRNAs were considered to be a consequence of the evolution of multicellularization, but it was later discovered that the unicellular green alga (*Chlamydomonas reinhardtii*) also encodes miRNAs (Molnar et al., 2007; Zhao et al., 2007), suggesting that the miRNAs pathway evolved prior to the divergence between unicellular algae and land plants. Moreover, most miRNA families in *Arabidopsis* have homologs in other plants, and several miRNA–mRNA target pairs are consistently conserved in primitive multicellular land plants (Bartel and Bartel, 2003; Jones-Rhoades, 2012; Zhang et al., 2013), suggesting that the miRNA has an ancient origin.

Three main models for the emergence and evolution of *MIR* genes in plant genomes have been suggested (Voinnet, 2009; Zhao et al., 2015; Zhang Y. et al., 2016). First, miRNAs are generated from the inverted duplication events of their target gene sequences (Allen et al., 2004; Maher et al., 2006); second, miRNAs originate from a variety of small-to-medium sized fold-back sequences distributed throughout the genome, termed ‘spontaneous evolution’ (Felippes et al., 2008); and third, DNA-type non-autonomous elements, namely miniature inverted-repeat transposable elements (MITEs) can readily fold into imperfect stem–loop structures of miRNA precursors (Piriyapongsa and Jordan, 2008). Because all life forms must survive their corresponding viruses, it is conceivable that host antiviral systems are essential in all living organisms (Villarreal, 2011). Indeed, viruses are crucial in the origin and evolution of host antiviral systems (Villarreal and Witzany, 2010; Villarreal, 2011). Although plant DNA viruses such as pararetroviruses and geminiviruses generally form episomal minichromosomes, illegitimate integration of these viruses in the plant genome is well documented (Hohn et al., 2008; Ghoshal and Sanfacon, 2015). Studies have also shown that cDNA sequences of plant RNA viruses can integrate into plant genomes, although plant RNA viruses are normally replicated in the cytoplasm of the infected cells (Hohn et al., 2008; Chiba et al., 2011). In addition, somatic endogenization may occur frequently, although it remains undetected because it is not passed on to the next generation (Covey and Al-Kaff, 2000). Remarkably, 24-nt sRNAs derived from an endogenous pararetrovirus sequence were found to accumulate to high levels in *Fritillaria imperialis* L. plants (Becher et al., 2014). Therefore, plant miRNAs may originate from viruses, such as virus-encoded miRNAs or miRNAs derived from the viral genome that integrated into the host genome. Two studies suggest the existence of virus-encoded miRNAs that may have been derived from *Sugarcane streak mosaic virus* (SCSMV) and *Hibiscus chlorotic ringspot virus* (HCRSV), respectively, but their functions remain to be elucidated (Gao et al., 2012; Viswanathan et al., 2014). In contrast, virus-encoded miRNAs have been identified extensively and are critical regulators of gene expression in animal–virus interactions (Nair and Zavolan, 2006; Grundhoff and Sullivan, 2011; Wang and Smith, 2016). However, more evidence is needed for the existence of plant virus–derived miRNAs.

## miRNAs and Plant Antiviral Defense

The successful survival of plants crucially depends upon their ability to exploit numerous defense mechanisms against invading pathogens or hostile environments. siRNA-mediated gene silencing is one of the most important strategies of plants against viral infections (Wang et al., 2012; Pumplin and Voinnet, 2013; Ghoshal and Sanfacon, 2015; Moon and Park, 2016; Khalid et al., 2017). There are two main advantages of siRNA-mediated gene silencing: the defensive signal can spread, and siRNA is transitive (Lu et al., 2008; Eamens et al., 2008). However, siRNA-mediated gene silencing is triggered only after viruses have invaded the host, thus infected cells are unable send a warning message to non-infected cells until the initial attack by viruses. Therefore, siRNA-mediated gene silencing may be insufficient to resist invading viruses, and a proactive mechanism is necessary. miRNAs are endogenous RNAs, some of miRNAs which exist within a cell prior to viral invasion while some miRNAs are induced previously in response to other stimuli or pathogens, indicating that these miRNAs can serve as advance preparation to counteract or evade the invading virus (Lu et al., 2008). Plant miRNAs have evolved to optimize cleavage efficiency rather than maximize complementarily to their targets (Voinnet, 2009; Jones-Rhoades, 2012). Three or more mismatches are permitted between miRNA and its target, which thereby significantly expands the spectrum of targets and facilitates the release of the cleaved target RNAs from the RISC complex. In plants, two main modes have been suggested for the roles of miRNAs in an antiviral defense response: a direct mode through targeting viral RNAs, and an indirect mode through triggering the biogenesis of siRNA responsible for the antiviral response.

Endogenous miRNAs have been shown to play an important role in the suppression of invading viruses in mammals (Gottwein and Cullen, 2008). In plants, miR393 was the first endogenous miRNA recognized to function in antibacterial resistance by suppressing auxin signaling (Navarro et al., 2006). In the same year, Simon-Mateo and Garcia (2006) demonstrated that *Plum pox virus* (PPV) chimeras bearing plant miRNA target sequences, which have been reported to be functional in *Arabidopsis*, were affected by miRNA function in three different host plants (Simon-Mateo and Garcia, 2006). In addition, several studies have shown that miRNA-mediated post-transcriptional regulation is involved in plant defensive responses against viral infections (Amin et al., 2011; Li et al., 2012; Pacheco et al., 2012). A recent study showed that cotton plants can export miRNAs to inhibit virulence gene expression in the fungal pathogen *Verticillium dahlia* (Zhang T. et al., 2016). The authors found that two genes encoding a Ca^2+^-dependent cysteine protease (*Clp-1*) and an isotrichodermin C-15 hydroxylase (*HiC-15*) targeted by miR166 and miR159, respectively, are both indispensable for *V. dahlia* virulence. Nevertheless, most studies provide indirect evidence for the first mode of plant miRNA function being direct targeting of viral RNAs, and more studies are needed to clarify this mode of action.

**FIGURE 2:**
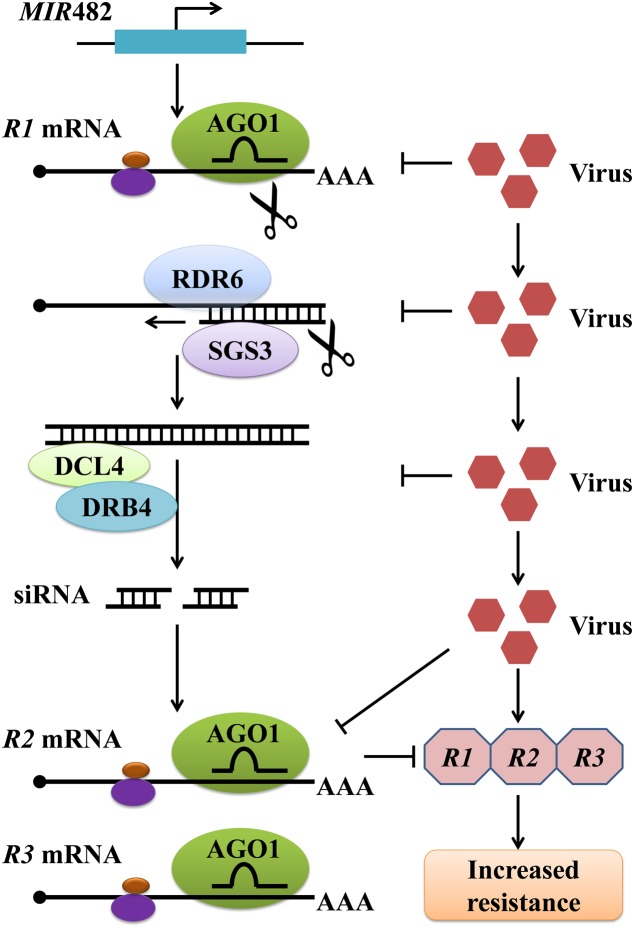
A pathway of plant miRNA482-mediated resistance against virus by inducing the production of siRNAs responsible for regulating *R* genes. The expression of *MIR*482 triggers the simultaneous silencing of multiple *R* genes through tasiRNAs produced from dsRNA derived from a primary miR482-targeted *R* gene. Virus infection may trigger silencing suppression involved in this process at several steps, resulting in increased accumulation of multiple *R* proteins and enhanced resistance. AGO1, Argonaute 1; DCL4, Dicer-like 4; RDR6, RNA-dependent RNA polymerase 6; SGS3, Suppressor of gene silencing 3; DRB4, Double-stranded RNA binding protein 4.

Plant genomes contain a large number of leucine-rich repeat (LRR) and nucleotide binding (NB)-LRR immune receptors encoded by resistance (*R*) genes, which recognize specific pathogen effectors and trigger resistance responses. To a great extent, the siRNA-mediated gene silencing involved in antiviral defense occurs through regulation of these *R* genes. Studies have shown that plant miRNAs target and negatively regulate plant *R* genes by prompting the production of phased, *trans*-acting siRNAs (tasiRNAs) against these *R* genes, and this miRNA-mediated gene regulation is suppressed on bacterial or viral infection (Zhai et al., 2011; Li et al., 2012). In *Medicago truncatula*, these ‘anti-*R* gene’ siRNAs are produced from dsRNA with the assistance of RNA-dependent RNA polymerase 6 (RDR6), DCL4, and DRB4 following the cleavage of certain *R* gene transcripts by miR482, a scheme that is similar to that of tasiRNA production (Zhai et al., 2011) (**Figure 2**). In tomato, miR482 can target a conserved sequence from 58 coiled coil (CC)-NB-LRR proteins, resulting in cleavage of *R* gene mRNA and production of secondary siRNAs in an RDR6-dependent manner (Shivaprasad et al., 2012). In tobacco, the *R* gene *N* against TMV, the first *R* gene conferring resistance to a virus to be identified, was found to undergo regulation by miR482 (Whitham et al., 1994; Li et al., 2012). In total, the silencing of NBS-LRR genes by miR482, and their activation after miR482 down-regulation upon bacterial or viral treatments, have been widely studied in different plants (Li et al., 2012; Shivaprasad et al., 2012; Zhu et al., 2013; Yang et al., 2015). Similarly, Li et al. (2012) demonstrated that miR6019 and miR6020 in tobacco cause specific cleavage of transcripts of the *N* gene and its homologs by binding to the complementary sequence of the conserved Toll and Interleukin-1 receptors (TIR)-encoding domain of the N transcript (Li et al., 2012; Moon and Park, 2016). Moreover, synthesis of phased, secondary siRNAs (phasiRNAs) from the *N* coding sequence through overexpression of miR6019 was shown to be accompanied by reductions in *N* transcript accumulation and *N*-mediated resistance against TMV (Li et al., 2012). Taken together, these results suggest that the miRNA-mediated gene silencing response is integrated with *R* gene–mediated antiviral defense responses.

## miRNAs and Viral Counter-Defense

Viruses have evolved numerous strategies to counteract or evade host defenses mediated by RNA silencing, such as the deployment of decoy RNAs, specialized replication mechanisms, and sequestration of viral RNAs in large protein or membrane complexes (Ghoshal and Sanfacon, 2015; Nie and Molen, 2015). Almost all plant viruses encode viral suppressors of RNA silencing (VSRs), which in addition to their functions in viral replication, encapsidation, or movement, interfere with host RNA silencing through multiple modes of action (Burgyan and Havelda, 2011; Wang et al., 2012). VSRs contribute to viral symptoms in two main ways: facilitating virus accumulation indirectly and modifying endogenous siRNA- or miRNA-mediated regulation directly (Silhavy and Burgyan, 2004; Burgyan and Havelda, 2011). In general, most VSR-mediated inhibition of RNA silencing occurs through two modes of action: (1) some VSRs sequester small RNA duplexes by binding to short or long dsRNAs, resulting in the suppression of the assembly of AGOs into RISCs; (2) some VSRs physically interact with AGO1 to prevent siRNA or miRNA loading, impede slicing activity, or degrade the AGO1 protein (Burgyan and Havelda, 2011; Wang et al., 2012; Moon and Park, 2016).

**FIGURE 3:**
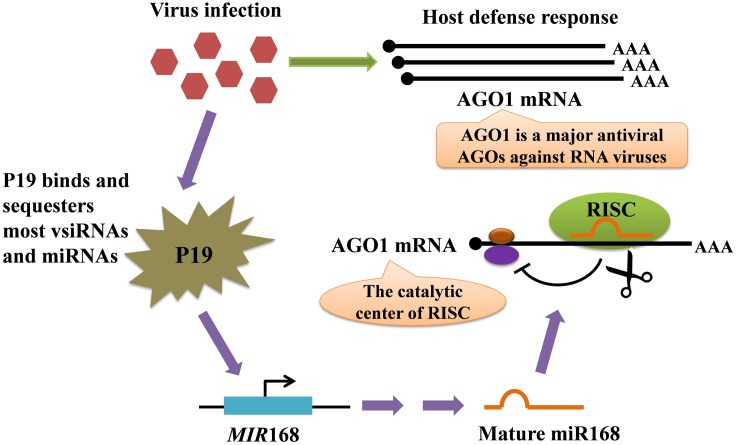
Model for the regulation of the AGO1 mRNA level mediated by tombusviral protein P19-induced miR168. The AGO1 protein plays a central role in plant defensive response against pathogens, and viral infection induces enhanced expression of AGO1 mRNA. Meanwhile, the virus produces the P19 VSR. P19 forms head-to-tail homodimers that bind to virus-encoded siRNAs (vsiRNAs), siRNAs and endogenous miRNAs with high affinity, preventing their loading into AGO1. However, miR168 is not efficiently bound by P19, resulting in the increased loading of miR168 into AGO1. Because miR168 directly represses the AGO1 mRNA, the accumulation of antiviral AGO1 is sharply decreased. In addition, tombusvirus infection also stimulates *MIR*168 transcription, and the expression of AGO1 mRNA is consequently further repressed by the increased miR168 level. Therefore, P19 VSR can not only sequester small RNAs, but can also effectively inhibit the loading of viral siRNAs onto AGO1.

The molecular basis of viral symptom development depends upon the ability of VSRs to interfere with plant miRNA biogenesis, eventually affecting mRNA turnover to the advantage of invaders (Chapman et al., 2004; Chen et al., 2004; Ramesh et al., 2014). The tombusvirus P19 protein is one of the best-studied VSRs that play critical roles in plant–virus interactions (Omarov et al., 2006; Várallyay et al., 2010) (**Figure 3**). The P19 binds and sequesters most miRNAs and virus-derived siRNAs (vsiRNAs) to suppress their activity in AGO proteins but is selectively unable to bind miR168, resulting in the increased loading of miR168 into AGO1 and the subsequent reduced accumulation of AGO1. Because miR168 directly down-regulates AGO1 mRNA stability and translation, this selective binding process not only causes the direct siRNA sequestration by P19 but also sharply reduces the cellular AGO1 levels (Várallyay et al., 2010; Pumplin and Voinnet, 2013). Tombusvirus infection also stimulates *MIR*168 transcription in a silencing inhibition–dependent manner, resulting in further increased levels of miR168 responsible for AGO1 down-regulation. Similar results have been observed during infections by other viruses, supporting that diverse VSRs convergently arrest endogenous silencing against the antiviral silencing pathway (Várallyay and Havelda, 2013). Notably, *African cassava mosaic virus* (ACMV) AC4, has been shown to bind directly to certain miRNAs, thereby making mi-RISC non-functional, and thus AC4 over-expressing transgenic plants showed reduced accumulation of miRNAs (Chellappan et al., 2005). Similarly, it is possible that *Tomato leaf curl new delhi virus* (ToLCNDV) AC4 might act to destabilize miRNAs which explains the reduction in the levels of certain miRNAs (Naqvi et al., 2010). In addition, a study demonstrated that *Rice stripe virus* (RSV) infections influenced small RNA profiles in rice, and that RSV induced the expression of novel miRNAs from conserved miRNA precursors (Du et al., 2011). These results suggest that VSRs and viral infection lead to major changes in the miRNA-mediated gene silencing pathway in plants.

Alternatively, some VSRs inhibit the activity of AGO proteins that have a central role in the antiviral RNA silencing (Wang et al., 2012; Carbonell and Carrington, 2015). For instance, *Sweet potato mild mottle virus* (SPMMV) P1 and *Turnip crinkle virus* (TCV) coat protein (CP or P38), directly interact with AGO proteins through conserved GW/WG repeat motifs, which resemble the AGO1-binding peptides on RISC (Giner et al., 2010; Moon and Park, 2016). In addition, Duan et al. (2012) demonstrated that *Cucumber mosaic virus* (CMV) 2b protein suppresses the activity of RISC by physically interacting with the PAZ domain of AGO1. These observations suggest that VSR suppression of RNA silencing may be associated with independently evolved VSRs that show functional overlap (Moon and Park, 2016).

Although some viruses can specifically disable host defense through encoding proteins, most viruses harbor limited coding capacity. Thus, the miRNAs become efficient and accessible tools to regulate their own gene expression and that of their host cells (Sullivan and Ganem, 2005; Nair and Zavolan, 2006). The first virus–encoded miRNAs were identified from a cloning experiment in human B cells latently infected with the herpesvirus Epstein-Barr virus (EBV) (Pfeffer et al., 2004). Subsequently, hundreds of animal virus–encoded miRNAs were discovered in various viruses such as herpesviruses, polyomaviruses, and adenoviruses (Gottwein and Cullen, 2008). Some animal virus–encoded miRNAs can effectively regulate viral gene expression and modulate the host’s miRNA-mediated gene silencing (Pfeffer et al., 2004; Nair and Zavolan, 2006; Roberts et al., 2011). During the counter-defense response, these animal virus–encoded miRNAs facilitate infection by regulating virus gene expression to increase virulence (Lu et al., 2008; Pumplin and Voinnet, 2013; Huang et al., 2016). The targets of viral miRNAs might be viral mRNAs or host cellular mRNAs, suggesting that viruses can employ miRNAs to regulate the cellular environment to support the viral life cycle (Roberts et al., 2011). In plants, numerous virus–derived siRNAs (vsiRNAs) or viroid–related siRNAs have been identified, and they play diverse functions in plant–virus interactions (Shimura et al., 2011; Smith et al., 2011; Avina-Padilla et al., 2015; Huang et al., 2016). In contrast, little evidence supports the existence of plant virus–encoded miRNAs, although two studies have suggested that they do exist (Gao et al., 2012; Viswanathan et al., 2014). A potential explanation for why metazoan virus–encoded miRNAs exist, while plant virus–encoded miRNAs have yet to be uncovered, may depend on the mode of action of animal infecting viruses (Ramesh et al., 2014). In fact, most of the mammalian viruses known to encode miRNAs have much larger genomes than most plant viruses, and those genomes are DNA rather than RNA, which is the most common type of genomic material for plant viruses (Wang et al., 2012). Consequently, for viruses with RNA genomes it would be at a fitness disadvantage if they encoded regions that were prone to endonucleolytic cleavage by DCL proteins or other mechanisms (Grundhoff and Sullivan, 2011; Roberts et al., 2011). The DNA viruses known to encode miRNAs replicate in the nucleus, while most plant viruses typically replicate in the cytoplasm where a miRNA precursor would be more exposed to cleavage that would likely inhibit replication of the virus carrying it as part of its genome (Grundhoff and Sullivan, 2011; Wang et al., 2012). Therefore, based on the requirements of nuclear machinery and RNA cleavage for miRNA processing, it is unsurprising that cytoplasmic replicating DNA viruses and RNA viruses have not been found to express miRNAs (Boss and Renne, 2011). Nevertheless, detection of both viral strands of *Turnip mosaic virus* (TuMV) within the nucleus showed that RNA viruses do enter the nucleus (Ramesh et al., 2014). In addition, some plant DNA viruses have been identified, such as Geminiviridae and Nanoviridae with DNA genomes which replicate through a dsDNA replicative intermediate (Hohn and Vazquez, 2011).

## miRNAs Involved in the Co-Evolution of Plants and Viruses

**FIGURE 4:**
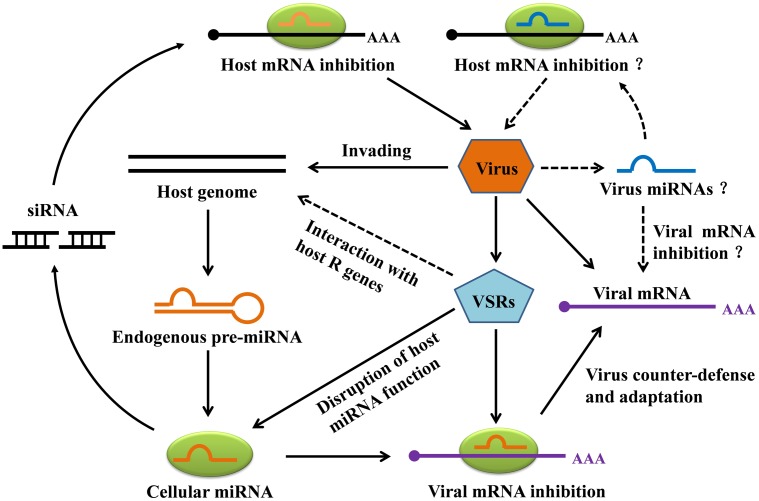
Hypothetical model for miRNA-mediated gene silencing in plant-virus interaction. Invading viruses can trigger the production of plant endogenous miRNAs. In addition to specifically repressing the expression of host genes, the miRNAs can target viral mRNA for degradation. To counteract host miRNA-mediated gene silencing, viruses most notably express viral suppressors of RNA silencing (VSRs) to avoid host RNA silencing. VSRs can not only interfere with host miRNA function, but can also repress naturally occurring silencing of host *R* genes. Although plant virus-derived miRNAs have not yet been discovered, we illustrate hypothetical viral strategies, including miRNA-mediated repression of the host and viral transcripts.

During the course of evolution, plants have evolved diverse strategies to counteract viral infection. Viruses have in turn evolved multiple mechanisms to counteract silencing, most obviously through the expression of VSRs. Interestingly, plants have also evolved specific defenses against RNA-silencing suppression by pathogens (Pumplin and Voinnet, 2013; Sansregret et al., 2013). The involvement of miRNAs in the never-ending arms race between plants and viruses has been summarized in **Figure 4**. As has been shown, some plant endogenous miRNAs can inhibit the expression of the plant’s own genes against invading viruses, and in addition some plant miRNAs can facilitate viral mRNA cleavage or inhibit viral mRNA translation. In the viral counter-defense mechanism, VSRs can efficiently inhibit host antiviral responses by interacting with host *R* genes, which are regulated by one or multiple miRNAs that are responsible for cellular silencing machinery. Also noteworthy here is a direct interaction between VSR and *R*-mediated defense that appears to be independent of the host RNA silencing pathways (Wang et al., 2012). For instance, the CMV 2b VSR suppressed salicylic acid–mediated defense response (Ji and Ding, 2001) while the HC-Pro VSR of *Potato virus Y* (PVY) was found to induce defense responses (Shams-Bakhsh et al., 2007), indicating that some VSRs are recognized by the host defense mechanism to induce antiviral resistance. In addition, **Figure 4** also illustrates a hypothesis that plant virus–derived miRNAs can inhibit viral mRNA, host mRNA, or both, though this remains to be verified.

In fact, miRNA-mediated gene silencing provides a selective force in shaping plant viral genomes (Ramesh et al., 2014; Ghoshal and Sanfacon, 2015). Additionally, the selective pressure of being targeted by host–encoded miRNAs and the ability of virus–encoded miRNA to target host genes may also have greatly contributed to the evolution of viral genomes (Wang et al., 2012; Incarbone and Dunoyer, 2013). Single nucleotide polymorphisms (SNPs) that inhibit viral miRNA-directed silencing of certain host genes may be positively selected in the viral genome. Likewise, sequence variations of the viral genome that prevent viruses from being targeted by host-encoded miRNAs might also be under positive selection during evolution. Viruses exist as mixtures of minor sequence variants, and their replication has a relatively high error rate. The rapid evolution of the viral genome may have contributed enormously to minimizing host miRNA-directed gene silencing in facilitating viral infection in a specific plant–virus interaction. An observation was that the viral genome can evolve rapidly against the suppression of host-derived miRNAs in PPV chimeras containing genomic miRNA target sites (Simon-Mateo and Garcia, 2006). Similarly, the evolutionary stability of amiRNA-mediated resistance against TuMV was evaluated by experiments, revealing that TuMV evade RNA silencing by rapidly accumulating mutations in the target regions (Lin et al., 2009). However, variations in a plant genome caused by viral infection can also contribute positively to its genome evolution by increasing genetic and epigenetic diversity. Notably, virus infections of endemic vegetation typically induce only mild symptoms, or the infections are latent, presumably as a result of co-evolution and selection of viruses that do not kill or seriously harm their hosts, and may even induce systemic acquired resistance against other pathogens (Lovisolo et al., 2003; Fraile and García-Arenal, 2010). In a sense, viruses are not just harmful pathogens, but also beneficial symbionts of plants (Villarreal, 2011). The co-evolution of pathogens and their hosts thereby facilitates the production of diverse sRNAs. Overall, miRNAs play diverse roles in plant defensive systems, but their functions in antiviral defense are far from being completely elucidated.

## The Application of miRNAs in Plant–Virus Interactions

Versatile plant biotechnologies, including antisense suppression, transcriptional gene silencing (TGS), virus-induced gene silencing (VIGS) and RNA interference (RNAi), are currently being used in plant antiviral biotechnology. In addition, artificial miRNA (amiRNA) is another robust biotechnology used in plants for silencing of genes, and engineering of amiRNAs has been widely applied for the targeted down-regulation of endogenous genes in various plants (**Table 1**). Given its efficacy and reliability, host-derived endogenous precursor miRNA has been commonly used as a structural backbone to replace the original ∼21 nt long miRNA sequence with a region complementary to the target viral genome (Schwab et al., 2006; Ramesh et al., 2014; Khalid et al., 2017). The PPV was modified to include *Arabidopsis* miRNA target sequences, and the engineered virus had clearly impaired infectivity due to *Nicotiana clevelandii* and *Nicotiana benthamiana* miRNA, although the behaviors of PPV chimeras vary in different plants (Simon-Mateo and Garcia, 2006). Multiple-target miRNAs can also simultaneously influence several viruses. For instance, miRNA precursors containing complementary sequences with *Turnip yellow mosaic virus* (TYMV) and TuMV were designed, and the transgenic *Arabidopsis* expressing the recombinant miRNA precursors displayed specific resistance to these viruses (Niu et al., 2006; Ai et al., 2011). In wheat, Fahim et al. (2012) developed an amiRNA strategy against *Wheat streak mosaic virus* (WSMV) by incorporating five amiRNAs within one polycistronic amiRNA precursor. These designed amiRNAs replaced the natural miRNAs in each of the five arms of the polycistronic rice miR395, producing an amiRNA precursor known as *FanGuard* (FGmiR395), which was transformed into wheat, leading to the transgenic plants resistance to WSMV. Recently, Sun et al. (2016) constructed three dimeric amiRNA precursor expression vectors that target the 3-proximal part of *CP* genes of RSV and *Rice black streaked dwarf virus* (RBSDV) based on the structure of the rice osa-MIR528 precursor. The transgenic rice plants showed high resistance simultaneously against RSV and RBSDV infection at a low temperature (Sun et al., 2016). Thus far, engineering of amiRNA for antiviral resistance has been used successfully in various plant species, including *N. benthamiana* (Qu et al., 2007; Ai et al., 2011; Kung et al., 2012; Ali et al., 2013; Song et al., 2014; Mitter et al., 2016; Wagaba et al., 2016; Carbonell and Daros, 2017), *Arabidopsis* (Duan et al., 2008; Lin et al., 2009), rice (Sun et al., 2016), wheat (Fahim et al., 2012), maize (Xuan et al., 2015), tomato (Zhang et al., 2011; Vu et al., 2013), and grapevine (Jelly et al., 2012) (**Table 1**). Apart from being used in plant antiviral immune systems, engineering of amiRNA has been extensively applied in plant resistance against other pathogens such as bacteria (Navarro et al., 2006; Li et al., 2010; Boccara et al., 2014; Ma et al., 2014), and fungi (Liu et al., 2014; Ouyang et al., 2014; Xu et al., 2014). These studies indicate that plant amiRNA biotechnology could be of broad utility in increasing plant resistance against pathogens.

**Table 1:** Engineering of plant miRNA for antiviral immunity.

Plant species	MiRNA backbone	Virus	Target viral region/gene	Reference
*Arabidopsis thaliana*	*Arabidopsis* pre-miR159	TYMV,	P69,	Niu et al., 2006
		TuMV	HC-Pro (coat protein)	
*Nicotiana benthamiana*	*Arabidopsis* miR159a, miR167b, and miR171a	PPV	P1/HC-Pro	Simon-Mateo and Garcia, 2006
*Nicotiana benthamiana*	*Arabidopsis* pre-miR171a	CMV	2b viral gene	Qu et al., 2007
*Arabidopsis thaliana*	*Arabidopsis* pre-miR159	CMV	3′-UTR	Duan et al., 2008
*Arabidopsis thaliana, Nicotiana benthamiana*	*Arabidopsis* pre-miR159	TuMV	P69	Lin et al., 2009
*Nicotiana tabacum*	*Arabidopsis* miR159a,	PVY	HC-Pro,	Ai et al., 2011
	miR167b, and miR171a	PVX	TGBp1/p25 (p25)	
*Solanum lycopersicum*	*Arabidopsis* pre-miR159a	CMV	2a and 2b viral genes, 3′-UTR	Zhang et al., 2011
*Nicotiana benthamiana*	*Arabidopsis* pre-miR159a	WSMoV	Conserved motifs of L (replicase) gene (A, B1, B2, C, D, E, AB1E, B2DC)	Kung et al., 2012
*Triticum*	Rice miR395	WSMV	Conserved region	Fahim et al., 2012
*Vitis vinifera*	*Arabidopsis* pre-miR319a	GFLV	Coat protein (CP)	Jelly et al., 2012
*Nicotiana benthamiana*	Cotton pre-miR169a	CLCuBuV	V2 gene	Ali et al., 2013
*Solanum lycopersicum*	*Arabidopsis* pre-miR319a, Tomato pre-miR319a and pre-miR168a	ToLCV	The middle region of the AV1 (coat protein), the overlapping region of the AV1 and AV2 (pre-coat protein)	Vu et al., 2013
*Nicotiana benthamiana Nicotiana. tabacum*	*Arabidopsis* pre-miR319a	PVY	CI, NIa, NIb, CP	Song et al., 2014
*Zea mays*	Maize pre-miR159a	RBSDV	Conserved region	Xuan et al., 2015
*Nicotiana benthamiana*	Barley pre-miR171	WDV	Conserved region	Kis et al., 2016
*Oryza sativa*	Rice pre-miR528	RSV, RBSDV	Middle segment, 3′ end and 3′-UTR region of the CP gene	Sun et al., 2016
*Nicotiana benthamiana*	*Arabidopsis* pre-miR159a	CBSV, UCBSV	P1, P3, CI, Nib and CP	Wagaba et al., 2016
*Nicotiana benthamiana*	*Arabidopsis* pre-miR159a	TSWV	N, NSs	Mitter et al., 2016
*Nicotiana benthamiana*	Six amiRNAs	PSTVd	Structural domains	Carbonell and Daros, 2017


*TYMV, Turnip yellow mosaic virus (Potyviridae); TuMV, Turnip mosaic virus (Potyviridae); CMV, Cucumber mosaic virus (Bromoviridae); PPV, Plum pox virus (Potyviridae); PVY, Potato virus Y (Potyviridae); PVX, Potato virus X (Alphaflexiviridae); WSMoV, Watermelon silver mottle virus (Bunyaviridae); WSMV, Wheat streak mosaic virus (Potyviridae); GFLV, Grapevine fan leaf virus (Secoviridae); CLCuBuV, Cotton leaf curl Burewala virus (Geminiviridae); WDV, Wheat dwarf virus (Geminiviridae); RSV, Rice stripe virus (unassigned); RBSDV, Rice black streaked dwarf virus (Reoviridae); CBSV, Cassava brown streak virus (Potyviridae); UCBSV, Ugandan cassava brown streak virus (Potyviridae); TSWV, Tomato spotted wilt virus (Bunyaviridae); PSTVd, Potato spindle tuber viroid (Pospiviroidae); ToLCV, Tomato leaf curl virus (Geminiviridae)*.

Previous studies revealed that the efficiency of miRNA to target viral RNAs depends not only on their nature but also on their inserted positions or the local structures of the target mRNAs (Simon-Mateo and Garcia, 2006; Duan et al., 2008). The accessibility of target sequences for amiRNA silencing is a pivotal factor for consideration. An experimental approach was used to determine the accessible cleavage hotspots on viral RNA by comparing the viral-derived siRNAs from wild-type *Arabidopsis* with sRNAs derived from those of the DCL mutants. The target viral transcript is assessed for DCL susceptibility and the vulnerable region was identified, thereby antiviral amiRNAs could be deployed (Duan et al., 2008). It is intriguing that the miRNA-mediated gene silencing mechanism or processing can also be affected by the flanking sequence in addition to the miRNA itself. The reasonable explanation is that RNA folding influences the binding sites between miRNAs and their target sequences (Lafforgue et al., 2013; Liu et al., 2016). Therefore, the insertion sites and the flanking sequence should be carefully validated when amiRNA-mediated gene silencing is established.

Engineering of amiRNAs possesses several advantages, including fewer off-target effects, high RNA promoter compatibility, high stability *in vivo*, high accuracy and the ability to degrade target genes without affecting expression of other genes, heritability of phenotypes, and environmental biosafety (Lu et al., 2008; Ramesh et al., 2014; Tiwari et al., 2014). Nevertheless, using amiRNA has several problems: (1) broad-spectrum amiRNAs are intractable to devise owing to the high sequence divergence of plant viruses; (2) the durability of amiRNAs is a challenge if the amiRNA targets the non-conserved regions of plant viruses; (3) single amiRNA expressing transgenic plants under field conditions may be confronted with strong virus pressure, thereby the resistance of transgenic plants against viruses may not be sustained. Fortunately, considerable efforts have been made to overcome these obstacles. For example, Lafforgue and his colleagues established two alternative strategies to improve the effectiveness of amiRNA including the expression of two amiRNAs complementary to independent targets and the design of amiRNAs complementary to highly conserved RNA motifs in the viral genome (Lafforgue et al., 2013). In addition, polycistronic amiRNA-mediated resistance to WSMV was successfully and efficiently applied in wheat and barley, respectively (Fahim et al., 2012; Kis et al., 2016). Recently, the Plant Small RNA Maker Site (P-SAMS) tool^1^, which serves as a high-throughput platform for the high efficiency design of amiRNA and synthetic *trans*-acting small interfering RNAs (syn-tasiRNA), has been established (Fahlgren et al., 2016). Collectively, there is still a long way to go for amiRNA engineering, although great progress has been made.

## Conclusion

Increasing evidence has shown that miRNA-mediated gene silencing plays a critical role in plant resistance against invading viruses and other types of pathogens. Although much remains to be learned about the molecular mechanisms of miRNA-mediated gene silencing in plants, current understanding has already laid a foundation for developing molecular tools for crop improvements. Due to the multiple advantages of amiRNA-mediated gene silencing, it has emerged as a powerful technique and become one of the most important tools in genetic engineering. However, failure and inefficiency of amiRNA-mediated gene silencing have been observed in some instances, probably due to the lack of complete knowledge of miRNA processing procedures involving biochemical enzymes and miRNA recruiting machinery. Hence, understanding the overall mechanisms of miRNA biogenesis is critical, beginning with transcription initiation and extending to target gene cleavage or translational repression. In addition, elucidation of the molecular mechanisms underlying the interactions between plants and viruses with respect to miRNAs will enable us to more thoroughly obtain the benefits to be derived from the miRNA-mediated gene silencing mechanism. Future efforts should be directed not only at understanding how to explore the machinery of viruses in hijacking the host miRNA-mediated gene silencing, but also developing rapid and systemic amiRNA delivery strategies to integrate amiRNAs in the plant genome.

## Author Contributions

S-RL wrote the paper, J-JZ, C-GH, C-LW, and J-ZZ wrote and edited the paper.

## Conflict of Interest Statement

The authors declare that the research was conducted in the absence of any commercial or financial relationships that could be construed as a potential conflict of interest.

## References

[B1] Ai T. Zhang L. Gao Z. Zhu C. X. Guo X. (2011). Highly efficient virus resistance mediated by artificial microRNAs that target the suppressor of PVX and PVY in plants. *Plant Biol. (Stuttg).* 13 304–316. 10.1111/j.1438-8677.2010.00374.x 21309977

[B2] Ali I. Amin I. Briddon R. W. Mansoor S. (2013). Artificial microRNA-mediated resistance against the monopartite begomovirus Cotton leaf curl Burewala virus. *Virol. J.* 10:231 10.1186/1743-422X-10-231 PMC3765727 23844988

[B3] Allen E. Xie Z. Gustafson A. M. Sung G. H. Spatafora J. W. Carrington J. C. (2004). Evolution of microRNA genes by inverted duplication of target gene sequences in *Arabidopsis thaliana*. *Nat. Genet.* 36 1282–1290. 10.1038/ng1478 15565108

[B4] Amin I. Basavaprabhu L. P. Briddon R. W. Mansoo S. Fauquet C. M. (2011). Common set of developmental miRNAs are upregulated in *Nicotiana benthamiana* by diverse begomoviruses. *Virol. J.* 8:143 10.1186/1743-422X-8-143 PMC3072929 21447165

[B5] Avina-Padilla K. Martinez de la Vega O. Rivera-Bustamante R. Martinez-Soriano J. P. Owens R. A. Hammond R. W. (2015). In silico prediction and validation of potential gene targets for pospiviroid-derived small RNAs during tomato infection. *Gene* 564 197–205. 10.1016/j.gene.2015.03.076 25862922

[B6] Axtell M. J. (2013). Classification and comparison of small RNAs from plants. *Annu. Rev. Plant Biol.* 64 137–159. 10.1146/annurev-arplant-050312-120043 23330790

[B7] Bartel B. (2005). microRNA directing siRNA biogenesis. *Nat. Struct. Mol. Biol.* 12 569–571. 10.1038/nsmb0705-569 15999111

[B8] Bartel B. Bartel D. P. (2003). MicroRNAs: at the root of plant development? *Plant Physiol.* 132 709–717. 10.1104/pp.103.023630 12805599 PMC523861

[B9] Bartel D. P. (2004). MicroRNAs: genomics, biogenesis, mechanism, and function. *Cell* 116 281–297. 10.1016/S0092-8674(04)00045-5 14744438

[B10] Baulcombe D. (2004). RNA silencing in plants. *Nature* 431 356–363. 10.1038/nature02874 15372043

[B11] Becher H. Ma L. Kelly L. J. Kovarik A. Leitch I. J. Leitch A. R. (2014). Endogenous pararetrovirus sequences associated with 24 nt small RNAs at the centromeres of *Fritillaria imperialis* L. (Liliaceae), a species with a giant genome. *Plant J.* 80 823–833. 10.1111/tpj.12673 25230921

[B12] Boccara M. Sarazin A. Thiebeauld O. Jay F. Voinnet O. Navarro L. (2014). The Arabidopsis miR472-RDR6 silencing pathway modulates PAMP- and effector-triggered immunity through the post-transcriptional control of disease resistance genes. *PLOS Pathog.* 10:e1003883 10.1371/journal.ppat.1003883 PMC3894208 24453975

[B13] Bologna N. G. Voinnet O. (2014). The diversity, biogenesis, and activities of endogenous silencing small RNAs in Arabidopsis. *Annu. Rev. Plant Biol.* 65 473–503. 10.1146/annurev-arplant-050213-035728 24579988

[B14] Boss I. W. Renne R. (2011). Viral miRNAs and immune evasion. *Biochim. Biophys. Acta* 1809 708–714. 10.1016/j.bbagrm.2011.06.012 21757042 PMC3864029

[B15] Brodersen P. Sakvarelidze-Achard L. Bruun-Rasmussen M. Dunoyer P. Yamamoto Y. Y. Sieburth L. (2008). Widespread translational inhibition by plant miRNAs and siRNAs. *Science* 320 1185–1190. 10.1126/science.1159151 18483398

[B16] Burgyan J. Havelda Z. (2011). Viral suppressors of RNA silencing. *Trends Plant Sci.* 16 265–272. 10.1016/j.tplants.2011.02.010 21439890

[B17] Calil I. P. Fontes E. P. (2016). Plant immunity against viruses: antiviral immune receptors in focus. *Ann. Bot.* 119 711–723. 10.1093/aob/mcw200 PMC5604577 27780814

[B18] Carbonell A. Carrington J. C. (2015). Antiviral roles of plant ARGONAUTES. *Curr. Opin. Plant Biol.* 27 111–117. 10.1016/j.pbi.2015.06.013 26190744 PMC4618181

[B19] Carbonell A. Daros J. A. (2017). Artificial microRNAs and synthetic trans-acting small interfering RNAs interfere with viroid infection. *Mol. Plant Pathol.* 18 746–753. 10.1111/mpp.12529 28026103 PMC6638287

[B20] Chapman E. J. Prokhnevsky A. I. Gopinath K. Dolja V. V. Carrington J. C. (2004). Viral RNA silencing suppressors inhibit the microRNA pathway at an intermediate step. *Genes Dev.* 18 1179–1186. 10.1101/gad.1201204 15131083 PMC415642

[B21] Chellappan P. Vanitharani R. Fauquet C. M. (2005). MicroRNA-binding viral protein interferes with Arabidopsis development. *Proc. Natl. Acad. Sci. U.S.A.* 102 10381–10386. 10.1073/pnas.0504439102 16006510 PMC1177406

[B22] Chen J. Xiang W. L. Xie D. Peng J. R. Ding S. W. (2004). Viral virulence protein suppresses RNA silencing-mediated defense but upregulates the role of microRNA in host gene expression. *Plant Cell* 16 1302–1313. 10.1105/tpc.018986 15100397 PMC423217

[B23] Chiba S. Kondo H. Tani A. Saisho D. Sakamoto W. Kanematsu S. (2011). Widespread endogenization of genome sequences of non-retroviral RNA viruses into plant genomes. *PLOS Pathog.* 7:e1002146 10.1371/journal.ppat.1002146 PMC3136472 21779172

[B24] Covey S. N. Al-Kaff N. S. (2000). Plant DNA viruses and gene silencing. *Plant Mol. Biol.* 43 307–322. 10.1023/A:1006408101473 10999413

[B25] Djuranovic S. Nahvi A. Green R. (2012). miRNA-mediated gene silencing by translational repression followed by mRNA deadenylation and decay. *Science* 336 237–240. 10.1126/science.1215691 22499947 PMC3971879

[B26] Du P. Wu J. Zhang J. Zhao S. Zheng H. Gao G. (2011). Viral infection induces expression of novel phased microRNAs from conserved cellular microRNA precursors. *PLOS Pathog.* 7:e1002176 10.1371/journal.ppat.1002176 PMC3161970 21901091

[B27] Duan C. G. Fang Y. Y. Zhou B. J. Zhao J. H. Hou W. N. Zhu H. (2012). Suppression of Arabidopsis ARGONAUTE1-mediated slicing, transgene-induced RNA silencing, and DNA methylation by distinct domains of the Cucumber mosaic virus 2b protein. *Plant Cell* 24 259–274. 10.1105/tpc.111.092718 22247253 PMC3289565

[B28] Duan C. G. Wang C. H. Fang R. X. Guo H. S. (2008). Artificial MicroRNAs highly accessible to targets confer efficient virus resistance in plants. *J. Virol.* 82 11084–11095. 10.1128/JVI.01377-08 18768978 PMC2573272

[B29] Eamens A. Wang M. B. Smith N. A. Waterhouse P. M. (2008). RNA silencing in plants: yesterday, today, and tomorrow. *Plant Physiol.* 147 456–468. 10.1104/pp.108.117275 18524877 PMC2409047

[B30] Fahim M. Millar A. A. Wood C. C. Larkin P. J. (2012). Resistance to Wheat streak mosaic virus generated by expression of an artificial polycistronic microRNA in wheat. *Plant Biotechnol. J.* 10 150–163. 10.1111/j.1467-7652.2011.00647.x 21895944

[B31] Fahlgren N. Hill S. T. Carrington J. C. Carbonell A. (2016). P-SAMS: a web site for plant artificial microRNA and synthetic trans-acting small interfering RNA design. *Bioinformatics* 32 157–158. 10.1093/bioinformatics/btv534 26382195 PMC4681993

[B32] Felippes F. F. D. Schneeberger K. Dezulian T. Huson D. H. Weigel D. (2008). Evolution of *Arabidopsis thaliana* microRNAs from random sequences. *RNA* 14 2455–2459. 10.1261/rna.1149408 18952822 PMC2590950

[B33] Fraile A. García-Arenal F. (2010). The coevolution of plants and viruses: resistance and pathogenicity. *Adv. Virus Res.* 76 1–32. 10.1016/S0065-3527(10)76001-2 20965070

[B34] Gao R. Liu P. Wong S. M. (2012). Identification of a plant viral RNA genome in the nucleus. *PLOS ONE* 7:e48736 10.1371/journal.pone.0048736 PMC3498252 23155403

[B35] Ghoshal B. Sanfacon H. (2015). Symptom recovery in virus-infected plants: revisiting the role of RNA silencing mechanisms. *Virology* 479–480 167–179. 10.1016/j.virol.2015.01.008 25677651

[B36] Giner A. Lakatos L. Garcia-Chapa M. Lopez-Moya J. J. Burgyan J. (2010). Viral protein inhibits RISC activity by argonaute binding through conserved WG/GW motifs. *PLOS Pathog.* 6:e1000996 10.1371/journal.ppat.1000996 PMC2904775 20657820

[B37] Gottwein E. Cullen B. R. (2008). Viral and cellular microRNAs as determinants of viral pathogenesis and immunity. *Cell Host Microbe* 3 375–387. 10.1016/j.chom.2008.05.002 18541214 PMC3079432

[B38] Grundhoff A. Sullivan C. S. (2011). Virus-encoded microRNAs. *Virology* 411 325–343. 10.1016/j.virol.2011.01.002 21277611 PMC3052296

[B39] Hohn T. Richert-Poggeler K. R. Staginnus C. Harper G. Schwartzacher T. Teo C. H. (2008). “Evolution of integrated plant viruses,” in *Plant Virus Evolution* ed. Roossinck M. (Berlin: Springer) 10.1007/978-3-540-75763-4-4

[B40] Hohn T. Vazquez F. (2011). RNA silencing pathways of plants: silencing and its suppression by plant DNA viruses. *Biochim. Biophys. Acta* 1809 588–600. 10.1016/j.bbagrm.2011.06.002 21683815

[B41] Huang J. Yang M. Lu L. Zhang X. (2016). Diverse functions of small RNAs in different plant-pathogen communications. *Front. Microbiol.* 7:1552 10.3389/fmicb.2016.01552 PMC5048074 27757103

[B42] Incarbone M. Dunoyer P. (2013). RNA silencing and its suppression: novel insights from in planta analyses. *Trends Plant Sci.* 18 382–392. 10.1016/j.tplants.2013.04.001 23684690

[B43] Iwakawa H. O. Tomari Y. (2013). Molecular insights into microRNA-mediated translational repression in plants. *Mol. Cell* 52 591–601. 10.1016/j.molcel.2013.10.033 24267452

[B44] Iwasaki S. Kobayashi M. Yoda M. Sakaguchi Y. Katsuma S. Suzuki T. (2010). Hsc70/Hsp90 chaperone machinery mediates ATP-dependent RISC loading of small RNA duplexes. *Mol. Cell* 39 292–299. 10.1016/j.molcel.2010.05.015 20605501

[B45] Jelly N. S. Schellenbaum P. Walter B. Maillot P. (2012). Transient expression of artificial microRNAs targeting Grapevine fanleaf virus and evidence for RNA silencing in grapevine somatic embryos. *Transgenic Res.* 21 1319–1327. 10.1007/s11248-012-9611-5 22427113

[B46] Ji L. H. Ding S. W. (2001). The suppressor of transgene RNA silencing encoded by Cucumber mosaic virus interferes with salicylic acid-mediated virus resistance. *Mol. Plant Microbe Interact.* 14 715–724. 10.1094/MPMI.2001.14.6.715 11386367

[B47] Jones-Rhoades M. W. (2012). Conservation and divergence in plant microRNAs. *Plant Mol. Biol.* 80 3–16. 10.1007/s11103-011-9829-2 21996939

[B48] Khalid A. Zhang Q. Yasir M. Li F. (2017). Small RNA based genetic engineering for plant viral resistance: application in crop protection. *Front. Microbiol.* 8:43 10.3389/fmicb.2017.00043 PMC5253543 28167936

[B49] Kis A. Tholt G. Ivanics M. Varallyay E. Jenes B. Havelda Z. (2016). Polycistronic artificial miRNA-mediated resistance to Wheat dwarf virus in barley is highly efficient at low temperature. *Mol. Plant Pathol.* 17 427–437. 10.1111/mpp.12291 26136043 PMC6638354

[B50] Kung Y. J. Lin S. S. Huang Y. L. Chen T. C. Harish S. S. Chua N. H. (2012). Multiple artificial microRNAs targeting conserved motifs of the replicase gene confer robust transgenic resistance to negative-sense single-stranded RNA plant virus. *Mol. Plant Pathol.* 13 303–317. 10.1111/j.1364-3703.2011.00747.x 21929564 PMC6638711

[B51] Lafforgue G. Martinez F. Niu Q. W. Chua N. H. Daros J. A. Elena S. F. (2013). Improving the effectiveness of artificial microRNA (amiR)-mediated resistance against Turnip mosaic virus by combining two amiRs or by targeting highly conserved viral genomic regions. *J. Virol.* 87 8254–8256. 10.1128/JVI.00914-13 23698292 PMC3700214

[B52] Lee R. C. Feinbaum R. L. Ambros V. (1993). The C. elegans heterochronic gene lin-4 encodes small RNAs with antisense complementarity to lin-14. *Cell* 75 843–854. 10.1016/0092-8674(93)90529-Y 8252621

[B53] Li F. Pignatta D. Bendix C. Brunkard J. O. Cohn M. M. Tung J. (2012). MicroRNA regulation of plant innate immune receptors. *Proc. Natl. Acad. Sci. U.S.A.* 109 1790–1795. 10.1073/pnas.1118282109 22307647 PMC3277104

[B54] Li S. Liu L. Zhuang X. Yu Y. Liu X. Cui X. (2013). MicroRNAs inhibit the translation of target mRNAs on the endoplasmic reticulum in Arabidopsis. *Cell* 153 562–574. 10.1016/j.cell.2013.04.005 23622241 PMC3694718

[B55] Li Y. Zhang Q. Zhang J. Wu L. Qi Y. Zhou J. M. (2010). Identification of microRNAs involved in pathogen-associated molecular pattern-triggered plant innate immunity. *Plant Physiol.* 152 2222–2231. 10.1104/pp.109.151803 20164210 PMC2850012

[B56] Lin S. S. Wu H. W. Elena S. F. Chen K. C. Niu Q. W. Yeh S. D. (2009). Molecular evolution of a viral non-coding sequence under the selective pressure of amiRNA-mediated silencing. *PLOS Pathog.* 5:e1000312 10.1371/journal.ppat.1000312 PMC2642722 19247440

[B57] Liu J. Cheng X. Liu D. Xu W. Wise R. Shen Q. H. (2014). The miR9863 family regulates distinct Mla alleles in barley to attenuate NLR receptor-triggered disease resistance and cell-death signaling. *PLOS Genet.* 10:e1004755 10.1371/journal.pgen.1004755 PMC4263374 25502438

[B58] Liu S. R. Hu C. G. Zhang J. Z. (2016). Regulatory effects of cotranscriptional RNA structure formation and transitions. *Wiley Interdiscip. Rev. RNA* 7 562–574. 10.1002/wrna.1350 27028291

[B59] Llave C. Xie Z. Kasschau K. D. Carrington J. C. (2002). Cleavage of scarecrow-like mRNA targets directed by a class of Arabidopsis miRNA. *Science* 297 2053–2056. 10.1126/science.1076311 12242443

[B60] Lovisolo O. Hull R. Rösler O. (2003). Coevolution of viruses with hosts and vectors and possible paleontology. *Adv. Virus Res.* 62 325–379. 10.1016/S0065-3527(03)62006-3 14719368

[B61] Lu Y. D. Gan Q. H. Chi X. Y. Qin S. (2008). Roles of microRNA in plant defense and virus offense interaction. *Plant Cell Rep.* 27 1571–1579. 10.1007/s00299-008-0584-z 18626646

[B62] Ma C. Lu Y. Bai S. Zhang W. Duan X. Meng D. (2014). Cloning and characterization of miRNAs and their targets, including a novel miRNA-targeted NBS-LRR protein class gene in apple (Golden Delicious). *Mol. Plant* 7 218–230. 10.1093/mp/sst101 23880633

[B63] Maher C. Stein L. Ware D. (2006). Evolution of Arabidopsis microRNA families through duplication events. *Genome Res.* 16 510–519. 10.1101/gr.4680506 16520461 PMC1457037

[B64] Mitter N. Zhai Y. Bai A. X. Chua K. Eid S. Constantin M. (2016). Evaluation and identification of candidate genes for artificial microRNA-mediated resistance to tomato spotted wilt virus. *Virus Res.* 211 151–158. 10.1016/j.virusres.2015.10.003 26454192

[B65] Molnar A. Schwach F. Studholme D. J. Thuenemann E. C. Baulcombe D. C. (2007). miRNAs control gene expression in the single-cell alga *Chlamydomonas reinhardtii*. *Nature* 447 1126–1129. 10.1038/nature05903 17538623

[B66] Moon J. Y. Park J. M. (2016). Cross-talk in viral defense signaling in plants. *Front. Microbiol.* 7:2068 10.3389/fmicb.2016.02068 PMC5174109 28066385

[B67] Nair V. Zavolan M. (2006). Virus-encoded microRNAs: novel regulators of gene expression. *Trends Microbiol.* 14 169–175. 10.1016/j.tim.2006.02.007 16531046

[B68] Nakanishi K. (2016). Anatomy of RISC: how do small RNAs and chaperones activate Argonaute proteins? *Wiley Interdiscip. Rev. RNA* 7 637–660. 10.1002/wrna.1356 27184117 PMC5084781

[B69] Naqvi A. R. Haq Q. M. Mukherjee S. K. (2010). MicroRNA profiling of tomato leaf curl new delhi virus (tolcndv) infected tomato leaves indicates that deregulation of mir159/319 and mir172 might be linked with leaf curl disease. *Virol. J.* 7:281 10.1186/1743-422X-7-281 PMC2972279 20973960

[B70] Navarro L. Dunoyer P. Jay F. Arnold B. Dharmasiri N. Estelle M. (2006). A Plant miRNA contributes to antibacterial resistance by repressing auxin signaling. *Science* 312 436–439. 10.1126/science.aae0382 16627744

[B71] Nie X. Molen T. A. (2015). Host recovery and reduced virus level in the upper leaves after Potato virus Y infection occur in tobacco and tomato but not in potato plants. *Viruses* 7 680–698. 10.3390/v7020680 25679498 PMC4353910

[B72] Niu Q. W. Lin S. S. Reyes J. L. Chen K. C. Wu H. W. Yeh S. D. (2006). Expression of artificial microRNAs in transgenic *Arabidopsis thaliana* confers virus resistance. *Nat. Biotechnol.* 24 1420–1428. 10.1038/nbt1255 17057702

[B73] Omarov R. Sparks K. Smith L. Zindovic J. Scholthof H. B. (2006). Biological relevance of a stable biochemical interaction between the Tombusvirus-encoded P19 and short interfering RNAs. *J. Virol.* 80 3000–3008. 10.1128/JVI.80.6.3000-3008.2006 16501109 PMC1395443

[B74] Ouyang S. Park G. Atamian H. S. Han C. S. Stajich J. E. Kaloshian I. (2014). MicroRNAs suppress NB domain genes in tomato that confer resistance to Fusarium oxysporum. *PLOS Pathog.* 10:e1004464 10.1371/journal.ppat.1004464 PMC4199772 25330340

[B75] Pacheco R. Garcıa-Marcos A. Barajas D. Martianez J. Tenllado F. (2012). PVX-potyvirus synergistic infections differentially alter microRNA accumulation in *Nicotiana benthamiana*. *Virus Res.* 165 231–235. 10.1016/j.virusres.2012.02.012 22387565

[B76] Pfeffer S. Zavolan M. Grasser F. A. Chien M. Russo J. J. Ju J. (2004). Identification of virus-encoded microRNAs. *Science* 304 734–736. 10.1126/science.1096781 15118162

[B77] Piriyapongsa J. Jordan I. K. (2008). Dual coding of siRNAs and miRNAs by plant transposable elements. *RNA.* 14 814–821. 10.1261/rna.916708 18367716 PMC2327354

[B78] Pumplin N. Voinnet O. (2013). RNA silencing suppression by plant pathogens: defence, counter-defence and counter-counter-defence. *Nat. Rev. Microbiol.* 11 745–760. 10.1038/nrmicro3120 24129510

[B79] Qu J. Ye J. Fang R. (2007). Artificial microRNA-mediated virus resistance in plants. *J. Virol.* 81 6690–6699. 10.1128/JVI.02457-06 17344304 PMC1900123

[B80] Ramesh S. V. Ratnaparkhe M. B. Kumawat G. Gupta G. K. Husain S. M. (2014). Plant miRNAome and antiviral resistance: a retrospective view and prospective challenges. *Virus Genes* 48 1–14. 10.1007/s11262-014-1038-z 24445902

[B81] Revers F. Nicaise V. (2014). “Plant resistance to infection by viruses,” in *Encyclopedia of Life Sciences* ed. Wiley-Blackwell (Chichester: John Wiley & Sons, Ltd) 10.1002/9780470015902.a0000757.pub3

[B82] Roberts A. P. Lewis A. P. Jopling C. L. (2011). The role of microRNAs in viral infection. *Prog. Mol. Biol. Transl. Sci.* 102 101–139. 10.1016/B978-0-12-415795-8.00002-7 21846570

[B83] Sansregret R. Dufour V. Langlois M. Daayf F. Dunoyer P. Voinnet O. (2013). Extreme resistance as a host counter-counter defense against viral suppression of RNA silencing. *PLOS Pathog.* 9:e1003435 10.1371/journal.ppat.1003435 PMC3681747 23785291

[B84] Schwab R. Ossowski S. Riester M. Warthmann N. Weigel D. (2006). Highly specific gene silencing by artificial microRNAs in Arabidopsis. *Plant Cell* 18 1121–1133. 10.1105/tpc.105.039834 16531494 PMC1456875

[B85] Shams-Bakhsh M. Canto M. Palukaitis P. (2007). Enhanced resistance and neutralization of defense responses by suppressors of RNA silencing. *Virus Res.* 130 103–109. 10.1016/j.virusres.2007.05.023 17617488

[B86] Shimura H. Pantaleo V. Ishihara T. Myojo N. Inaba J. Sueda K. (2011). A viral satellite RNA induces yellow symptoms on tobacco by targeting a gene involved in chlorophyll biosynthesis using the RNA silencing machinery. *PLOS Pathog.* 7:e1002021 10.1371/journal.ppat.1002021 PMC3088725 21573143

[B87] Shivaprasad P. V. Chen H. M. Patel K. Bond D. M. Santos B. A. Baulcombe D. C. (2012). A microRNA superfamily regulates nucleotide binding site-leucine-rich repeats and other mRNAs. *Plant Cell* 24 859–874. 10.1105/tpc.111.095380 22408077 PMC3336131

[B88] Silhavy D. Burgyan J. (2004). Effects and side-effects of viral RNA silencing suppressors on short RNAs. *Trends Plant Sci.* 9 76–83. 10.1016/j.tplants.2003.12.010 15102373

[B89] Simon-Mateo C. Garcia J. A. (2006). MicroRNA-guided processing impairs Plum pox virus replication, but the virus readily evolves to escape this silencing mechanism. *J. Virol.* 80 2429–2436. 10.1128/JVI.80.5.2429-2436.2006 16474149 PMC1395392

[B90] Smith N. A. Eamens A. L. Wang M. B. (2011). Viral small interfering RNAs target host genes to mediate disease symptoms in plants. *PLOS Pathog.* 7:e1002022 10.1371/journal.ppat.1002022 PMC3088724 21573142

[B91] Song Y. Z. Han Q. J. Jiang F. Sun R. Z. Fan Z. H. Zhu C. X. (2014). Effects of the sequence characteristics of miRNAs on multi-viral resistance mediated by single amiRNAs in transgenic tobacco. *Plant Physiol. Biochem.* 77 90–98. 10.1016/j.plaphy.2014.01.008 24561715

[B92] Sullivan C. S. Ganem D. (2005). MicroRNAs and viral infection. *Mol. Cell* 20 3–7. 10.1016/j.molcel.2005.09.012 16209940

[B93] Sun L. Lin C. Du J. Song Y. Jiang M. Liu H. (2016). Dimeric artificial microRNAs mediate high resistance to RSV and RBSDV in transgenic rice plants. *Plant Cell Tiss. Organ. Cult.* 126 127–139. 10.1007/s11240-016-0983-8

[B94] Tiwari M. Sharma D. Trivedi P. K. (2014). Artificial microRNA mediated gene silencing in plants: progress and perspectives. *Plant Mol. Biol.* 86 1–18. 10.1007/s11103-014-0224-7 25022825

[B95] Várallyay E. Havelda Z. (2013). Unrelated viral suppressors of RNA silencing mediate the control of ARGONAUTE1 level. *Mol. Plant Pathol.* 14 567–575. 10.1111/mpp.12029 23578299 PMC6638692

[B96] Várallyay E. Válóczi A. Agyi A. Burgyán J. Havelda Z. (2010). Plant virus-mediated induction of miR168 is associated with repression of ARGONAUTE1 accumulation. *EMBO J.* 29 3507–3519. 10.1038/emboj.2010.215 20823831 PMC2964164

[B97] Villarreal L. P. (2011). Viral ancestors of antiviral systems. *Viruses* 3 1933–1958. 10.3390/v3101933 22069523 PMC3205389

[B98] Villarreal L. P. Witzany G. (2010). Viruses are essential agents within the roots and stem of the tree of life. *J. Theor. Biol.* 262 698–710. 10.1016/j.jtbi.2009.10.014 19833132

[B99] Viswanathan C. Anburaj J. Prabu G. (2014). Identification and validation of sugarcane streak mosaic virus-encoded microRNAs and their targets in sugarcane. *Plant Cell Rep.* 33 265–276. 10.1007/s00299-013-1527-x 24145912

[B100] Voinnet O. (2009). Origin, biogenesis, and activity of plant microRNAs. *Cell* 136 669–687. 10.1016/j.cell.2009.01.046 19239888

[B101] Vu T. V. Choudhury N. R. Mukherjee S. K. (2013). Transgenic tomato plants expressing artificial microRNAs for silencing the pre-coat and coat proteins of a begomovirus, Tomato leaf curl New Delhi virus, show tolerance to virus infection. *Virus Res.* 172 35–45. 10.1016/j.virusres.2012.12.008 23276684

[B102] Wagaba H. Patil B. L. Mukasa S. Alicai T. Fauquet C. M. Taylor N. J. (2016). Artificial microRNA-derived resistance to Cassava brown streak disease. *J. Virol. Methods* 231 38–43. 10.1016/j.jviromet.2016.02.004 26912232 PMC4819903

[B103] Wang M. B. Masuta C. Smith N. A. Shimura H. (2012). RNA silencing and plant viral diseases. *Mol. Plant Microbe Interact.* 25 1275–1285. 10.1094/MPMI 22670757

[B104] Wang M. B. Smith N. A. (2016). Satellite RNA pathogens of plants: impacts and origins-an RNA silencing perspective. *Wiley Interdiscip. Rev. RNA* 7 5–16. 10.1002/wrna.1311 26481458

[B105] Whitham S. Dinesh-Kumar S. P. Choi D. Hehl R. Corr C. Baker B. (1994). The product of the tobacco mosaic virus resistance gene N: similarity to toll and the interleukin-1 receptor. *Cell* 78 1101–1115. 10.1016/0092-8674(94)90283-6 7923359

[B106] Xu W. Meng Y. Wise R. P. (2014). Mla- and Rom1-mediated control of microRNA398 and chloroplast copper/zinc superoxide dismutase regulates cell death in response to the barley powdery mildew fungus. *New Phytol.* 201 1396–1412. 10.1111/nph.12598 24246006

[B107] Xuan N. Zhao C. Peng Z. Chen G. Bian F. Lian M. (2015). Development of transgenic maize with anti-rough dwarf virus artificial miRNA vector and their disease resistance. *Chin. J. Biotechnol.* 31 1375–1386. 26955715

[B108] Yang L. Mu X. Liu C. Cai J. Shi K. Zhu W. (2015). Overexpression of potato miR482e enhanced plant sensitivity to *Verticillium dahliae* infection. *J. Integr. Plant Biol.* 57 1078–1088. 10.1111/jipb.12348 25735453

[B109] Zhai J. Jeong D. H. De Paoli E. Park S. Rosen B. D. Li Y. (2011). MicroRNAs as master regulators of the plant NB-LRR defense gene family via the production of phased, trans-acting siRNAs. *Genes Dev.* 25 2540–2553. 10.1101/gad.177527.111 22156213 PMC3243063

[B110] Zhang J. Z. Ai X. Y. Guo W. W. Peng S. A. Deng X. X. Hu C. G. (2012). Identification of miRNAs and their target genes using deep sequencing and degradome analysis in trifoliate orange [*Poncirus trifoliata* (L.) Raf]. *Mol. Biotechnol.* 51 44–57. 10.1007/s12033-011-9439-x 21796478

[B111] Zhang S. Yue Y. Sheng L. Wu Y. Fan G. Li A. (2013). PASmiR: a literature-curated database for miRNA molecular regulation in plant response to abiotic stress. *BMC Plant Biol.* 13:33 10.1186/1471-2229-13-33 PMC3599438 23448274

[B112] Zhang T. Zhao Y. L. Zhao J. H. Wang S. Jin Y. Chen Z. Q. (2016). Cotton plants export microRNAs to inhibit virulence gene expression in a fungal pathogen. *Nat. Plants* 2:16153 10.1038/nplants.2016.153 27668926

[B113] Zhang X. Li H. Zhang J. Zhang C. Gong P. Ziaf K. (2011). Expression of artificial microRNAs in tomato confers efficient and stable virus resistance in a cell-autonomous manner. *Transgenic Res.* 20 569–581. 10.1007/s11248-010-9440-3 20835923

[B114] Zhang Y. Xia R. Kuang H. Meyers B. C. (2016). The diversification of plant NBS-LRR defense genes directs the evolution of MicroRNAs that target them. *Mol. Biol. Evol.* 33 2692–2705. 10.1093/molbev/msw154 27512116 PMC5026261

[B115] Zhao M. Meyers B. C. Cai C. Xu W. Ma J. (2015). Evolutionary patterns and coevolutionary consequences of MIRNA genes and microRNA targets triggered by multiple mechanisms of genomic duplications in soybean. *Plant Cell* 27 546–562. 10.1105/tpc.15.00048 25747880 PMC4558674

[B116] Zhao T. Li G. Mi S. Li S. Hannon G. J. Wang X. J. (2007). A complex system of small RNAs in the unicellular green alga *Chlamydomonas reinhardtii*. *Genes Dev.* 21 1190–1203. 10.1101/gad.1543507 17470535 PMC1865491

[B117] Zhu Q. Fan L. Liu Y. Xu H. Llewellyn D. Wilson I. (2013). miR482 regulation of NBS-LRR defense genes during fungal pathogen infection in cotton. *PLOS ONE* 8:e84390 10.1371/journal.pone.0084390 PMC3877274 24391949

